# Increased HIV-1 pretreatment drug resistance with consistent clade homogeneity among ART-naive HIV-1 infected individuals in Ethiopia

**DOI:** 10.1186/s12977-020-00542-0

**Published:** 2020-09-29

**Authors:** Mulugeta Kiros, Dawit Hailu Alemayehu, Eleni Geberekidan, Adane Mihret, Melanie Maier, Woldaregay Erku Abegaz, Andargachew Mulu

**Affiliations:** 1 Department of Medical Laboratory Science, College of Medicine and Health Science, Debre Tabor University, Debre Tabor, Ethiopia; 2 Armauer Hansen Research Institute, Addis Ababa, Ethiopia; 3 The Ethiopian Public Health Institute, Addis Ababa, Ethiopia; 4 Institute of Virology, Leipzig University, Leipzig, Germany; 5 Department of Microbiology, Parasitology, and Immunology, School of Medicine, Addis Ababa University, Addis Ababa, Ethiopia

**Keywords:** HIV-1, HIV-1 genetic diversity, Pretreatment drug resistance, HIV-1 subtype, ART-naive, Ethiopia

## Abstract

**Background:**

The development of pretreatment drug resistance (PDR) is becoming an obstacle to the success of antiretroviral therapy (ART). Besides, data from developing settings including Ethiopia is still limited. Therefore, this study was aimed to assess HIV-1 genetic diversity and PDR mutations among ART-naive recently diagnosed HIV-1 infected individuals in Addis Ababa, Ethiopia.

**Methods:**

Institutional based cross-sectional study was conducted from June to December 2018 in Addis Ababa among ART-naive recently diagnosed individuals. Partial HIV-1 pol region covering the entire protease (PR) and partial reverse transcriptase (RT) regions of 51 samples were amplified and sequenced using an in-house assay. Drug resistance mutations were examined using calibrated population resistance (CPR) tool version 6.0 from the Stanford HIV drug resistance database and the International Antiviral Society-USA (IAS-USA) 2019 mutation list.

**Results:**

According to both algorithms used, 9.8% (5/51) of analyzed samples had at least one PDR Mutation. PDR mutations to Non-Nucleoside Reverse Transcriptase Inhibitors (NNRTIs) were the most frequently detected (7.8% and 9.8%, according to the CPR tool and IAS-USA algorithm, respectively). The most frequently observed NNRTIs-associated mutations common to both algorithms were K103N (2%), Y188L (2%), K101E (2%), and V106A (2%), while E138A (2%) was observed according to IAS-USA only. Y115F and M184V (mutations that confer resistance to NRTIs) dual mutations were detected according to both criteria in a single study participant (2%). PDR mutation to protease inhibitors was found to be low (only G73S; 2% according to the CPR tool). Phylogenetic analysis showed that 98% (50/51) of the study participants were infected with HIV-1C virus while one individual (2%) was infected with HIV-1A1 virus.

**Conclusions:**

This study showed an increased level of PDR and persistence HIV-1C clade homogeneity after 15 years of the rollout of ART and 3 decades of HIV-1C circulation in Ethiopia, respectively. Therefore, we recommend routine baseline genotypic drug resistance testing for all newly diagnosed HIV infected patients before initiating treatment. This will aid the selection of appropriate therapy in achieving the 90% of patients having an undetectable viral load in consonance with the UN target.

## Background

Human Immunodeficiency Virus (HIV) is a major global public health issue with millions of people infected worldwide [1]. Africa is the most affected region among other continents in the globe with the highest rise of the illness concentrating in the eastern and southern parts [2]. In Eastern and Southern Africa, which is the home of 54% of the world’s people living with HIV [3], there were an estimated 800,000 new HIV infections which account for around 47% of the total global new infection and 310,000 people dying from Acquired Immuno Deficiency Syndrome (AIDS)-related illness in the same year [3].

In Ethiopia, HIV/AIDS is among the top ten leading causes of age-standardized premature mortality and death [4]. In 2018, there were an estimated 23,000 new infections and 11,000 AIDS-related deaths [3]. The rate of new infections showed an increment from the previous year which was 16,000 new infections in 2017 [5]. There were an estimated 690,000 people living with HIV in the country in 2018 [3]. According to data from the Federal HIV/AIDS Prevention and Control Office, the HIV prevalence in the country in 2018 was 0.9% [6].

Although ART is contributing a lot in prolonging the life of HIV infected individuals [7], scaling up of this treatment option along with the absence of drug resistance testing in resource-limited settings is paralleled by an increased prevalence of pretreatment drug resistance (PDR) [8, 9]. This is becoming a significant obstacle in maintaining suppression of HIV replication leading to a higher probability of early virological failure [10] that further hinders the widespread use of ART [11, 12]. Likewise, it is becoming a potential threat to the long-term success of ART and is emerging as a threat to the elimination of AIDS as a public health problem [13].

According to the Ethiopian Ministry of Health updated guideline for HIV prevention, care and treatment, the current preferred first-line regimen combination for adults and adolescents is TDF + 3TC + DTG/EFV. The alternative drug combinations include AZT + 3TC + EFV or TDF + 3TC + NVP. AZT + 3TC + ATV/r or LPV/r are drug of choices in the second-line ART. In the third line treatment strategy, the combination is as follows; DRV/r +ABC + 3TC + EFVor NVP [14].

Although it has been 15 years since the rollout of ART in Ethiopia, only a few studies are done with regard to PDR with the majority of them being from the Northern part of Ethiopia [15–18] and data from the capital city is limited [19, 20]. Thus, this study was aimed at generating updated information about genetic diversity and magnitude of PDR in Addis Ababa that may be utilized in alleviating the problem of treatment failure and consequently reducing the burden of the disease.

## Methods

### Study subjects and study design

Institutional based cross-sectional study was conducted among 51 ART-naive HIV infected asymptomatic adult individuals in Addis Ababa. The study was conducted in four government-affiliated voluntary counseling test centers in Addis Ababa from June to December 2018. The sites were selected based on the availability of a high flow of individuals in their testing center. Study subjects who are asymptomatic, ≥ 18 years old, and willing to participate in this study were sequentially included in this study. Pregnant women, individuals with known chronic illnesses, or any previous ART use were excluded from this study. The sample size used in this study is concurrent with the WHO recommendation for the surveillance of HIVDR in resource-limited settings. According to the recommendation, it is advised to analyze sequences from a minimum of 47 individuals [21].

### Sample collection

A blood sample (10 ml) was collected by trained medical personnel aseptically. Vacutainer test tubes containing ethylene diamine tetra-acetic acid (EDTA) were used to collect the blood samples. The sample was labeled with appropriate patient information. Plasma was then separated from blood cells within 2 h of collection in the laboratory by centrifugation at the speed of 1200*g* (3000 rpm) for 10 to 15 min in accordance with the recommendation of WHO. The plasma was aliquotted into Nunc tubes of 1.5-ml capacity, and transported in an icebox on the same day to the AHRI laboratory, where it was stored at − 80 °C until required for molecular analysis.

### Laboratory investigations

#### Viral load and RNA extraction

HIV-1 RNA extraction and viral quantification were done using Abbott Real-time HIV-1 M2000rt extraction machine (Abbott Laboratories, Abbott Park, USA).

### Reverse transcription polymerase chain reaction (RT-PCR)

cDNA for the entire PR [99 codons] and partial RT [the first 308 codons] regions was synthesized in a 20 µl reaction mixture using superscript IV Reverse Transcriptase enzyme and HIVrt primer (5′-TGTTTTACATCATTAGTGTG-3′, HXB2 location: 3630–3649). The thermal cycling for cDNA synthesis was; 50 °C for 1 h [17]. After cDNA was synthesized, we used Platinum Taq High Fidelity (Invitrogen, Carlsbad, CA, USA) polymerase enzyme and two in-house outer primers (HIVpcrFor1: 5′-TGATGACAGCATGTCAGGGAGTGG-3′, HXB2 location 1826–1849 and HIVpcrRev1: 5′-GGCTCTTGATAAATTTGATATGTCCATTG-3′, HXB2 location 3555–3583) for the first-round PCR. The reaction was performed in a 50 µl reaction mixture and the cycling conditions used were as follows: initial denaturation at 94 °C for 2 min, 35 cycles of 30 s at 94 °C, 1 min at 54 °C, 1 min at 72 °C followed by a final extension for 5 min at 72 °C [17]. This yields 1757 bp amplicon (covering the entire protease [99 codons] and partial reverse transcriptase [the first 308 codons] regions).

### Nested PCR and DNA purification

Second-round PCR was performed to re-amplify an amplicon from first-round PCR. This was carried out using High-Fidelity Taq polymerase (Life Technologies, USA) and two inner in-house primers (HIVpcrFor2: 5′-AGCCAACAGCCCCACCAG-3′, HXB2 location 2150–2167 and HIVpcrRev2: 5′-CTGTATTTCTGCTATTAAGTCTTTTG-3′, HXB2 location 3514–3539). The reaction was performed in a 50 Âµl reaction mixture similar to the first-round PCR and the thermal cycling consisted of initial denaturation at 94 °C for 2 min, 35 cycles of 30 s at 94 °C, 1 min at 54 °C, 1 min at 72 °C followed by a final extension for 5 min at 72 °C [17]. Agarose gel electrophoresis (using 1.5% agarose gel) was then performed to confirm the final amplified PCR product (1389 bp; covering the entire protease region [99 codons] and partial reverse transcriptase [the first 308 codons of the RT region]) [17]. This was followed by DNA purification using GeneJET Gel Extraction and DNA Cleanup Micro Kit (Thermo Fisher Scientific, Inc., United States), following the manufacturer’s instruction. The Quality and concentration of purified DNA were checked using both NanoDrop and agarose gel electrophoresis.

### DNA sequencing

Cycle sequencing reaction (by Sanger sequencing method) for each purified DNA sample was performed using Big Dye Terminator Cycle Sequencing Ready Reaction mix v.3.1 (Applied Biosystems, USA) and four in-house inner primers (HIVpcrFor2: 5′-AGCCAACAGCCCCACCAG-3′, HXB2 location 2150–2167, HIVSeq 1: 5′-GTTAAACAATGGCCATTGACAGA-3′, HXB2 location 2610–2632, HIVSeq 4: 5′-CCATCCCTGTGGAAGCACATT-3′, HXB2 location 2988–3008, HIVpcrRev2: 5′-CTGTATTTCTGCTATTAAGTCTTTTG-3′, HXB2 location, 3514–3539). The thermal cycling consisted of initial denaturation at 96 °C for 2 min, 39 cycles of 30 s at 96 °C, 15 s at 56 °C, 5 min at 60 °C and a final extension for 5 min at 60 °C [17]. Following cycle sequencing, the excess dye terminators were removed using DyeEx 2.0 Spin Kit (Qiagen, Germany) following the manufacturer’s instruction. The analyte was further dried using vacuum centrifuge, and stored at − 20 °C at AHRI laboratory until sequenced. These dried DNA samples were then treated with 20 µl of formamide and subsequently processed with an automated ABI 3500 xL Genetic Analyzer (Applied Biosystems).

### Data processing and analysis

#### Data management and quality assurance

A structured questionnaire was used to collect socio-demographic data like age, sex, occupation, etc. of the study participants. The questionnaires were then checked for any errors and completeness of the response given by participants before data entry to ensure the quality of the result. In addition, visual inspection of the reagent bottles and expiration date checks were performed before each laboratory work. The laboratory procedures were conducted in separately designated laboratory spaces for quality control purposes. Both positive and negative controls were used for each laboratory work. Overall, standard operating procedures were strictly followed to ensure the quality and accuracy of the test result. Quality of sequence data was checked using an online data management and quality assurance tool found in the Los Alamos HIV sequence database (http://hiv-web.lanl.gov).

### Statistical analyses

Demographic and clinical data recorded from questionnaire responses of each participant (age, gender, etc.) were checked for completeness and entered into Epi data v3.1 software and exported to SPSS version 25.0 (SPSS Inc. the United States) for analysis. Logistic regression was used to assess the associations between PDR and demographic or virological characteristics. The PDR prevalence was determined with a confidence interval (CI) of 95%. A p-value < 0.05 was considered significant. Drug resistance mutations for PI, NRTI, and NNRTIs were characterized by their frequency and percentages.

### Sequence analysis

#### Sequence editing, alignment, subtype determination and phylogenetic analysis

SeqA5.4 software, which was contained in the ABI PRISM^®^ 3500 xL Genetic Analyzer (Applied Biosystems) collects, processes, and stores the data automatically after each run. Then sequences were exported to other computer and were first edited using chromas software v.2.6.6 (http://technelysium.com.au/wp/chromaspro/) and Geneious prime^®^ v.2019.2.1 (https://www.geneious.com/academic/). Then the fragment sequences were aligned using the latter software. Then HIV-1 subtype determination was done using the REGA HIV subtyping tool (Leuven University, Leuven, Belgium; https://www.genomedetective.com/app/typingtool/hiv) and were further confirmed by phylogenetic analysis using reference sequences from Los Alamos National Laboratory HIV Sequence Database (http://hiv-web.lanl.gov) (Fig. 2). The Geneious prime^®^ v.2019.2.1 software was used to draw neighbor-joining trees under the Tamura Nei genetic distance model. The statistical robustness of the neighbour-joining tree and reliability of the branching patterns were confirmed by bootstrap analysis (1000 replicates).

### Pretreatment drug resistance determination

PDR determination was performed using the Stanford Genotypic Resistance calibrated population resistance (CPR) tool version 6.0 contained in the Stanford HIVdb (http://StanfordHIVdb.stanford.edu) algorithm and the IAS-USA 2019 mutation list. Classification of PDR level (low: < 5%, moderate: 5–15%, or high: > 15%) was made based on the WHO threshold survey protocol [21].

## Results

### Sociodemographic characteristics of study participants

Partial HIV-1 pol sequence covering the complete PR [99 codons] and partial RT [the first 308 codons] regions of 51 study participants were successfully sequenced, analyzed, and submitted to Genbank (accession numbers MT416661–MT416711). Of those 51 studied subjects, 54.9% were female. The average age of study participants was 37 years. Most (80.4%) of the study participants reported having a secondary and/or less than secondary school attendance as shown in the table (Table 1). With regard to viral load, 41.2% of study participants had 100,001–500,000 copies/ml (Table 1). No significant associations were observed between participants’ demographic characteristics and Pretreatment Drug Resistance Mutations (PDRMs) (Table 1).

**Table 1:** Sociodemographic and virological characteristics of included study participants

Characteristics	Frequency (N)	Percentage (%)	Individuals with PDRM (N)	
			Based on CPR tool	Based on IAS-USA
Sex				
Male	23	45.1	2	2
Female	28	54.9	3	3
Age category				
18–28	23	45.1	1	2
29–38	17	33.3	2	1
39–48	8	15.7	2	2
> 49	3	5.9	0	0
Baseline viral load (copies/ml)				
2000–10,000	2	3.9	0	0
10,001–100,000	19	37.3	2	3
100,001–500,000	21	41.2	3	2
> 500,000	9	17.6	0	0
Occupation				
Unemployed	31	60.8	3	4
Employed	20	31.2	2	1
Marital status				
Married	19	37.3	1	1
Single	14	27.5	0	1
Divorced	14	27.5	4	3
Widowed/widower	4	7.8	0	0
Educational status				
No schooling	8	15.7	0	
Primary	15	25.4	1	2
Secondary	18	35.3	2	2
College (diploma)	3	5.9	1	1
University degree	7	13.7	1	0

### The magnitude of pretreatment drug resistance

According to both the CPR tool and IAS-USA algorithms, 9.8% (5/51, CI (3.3–21.4)) of analyzed samples had at least one PDR Mutation. PDR mutations to NNRTIs were the most frequently detected mutations (4/51; 7.8%, CI (2.2–18.9) and 5/51; 9.8%, CI (3.3–21.4)) according to the CPR tool and IAS-USA mutation list, respectively) followed by NRTIs (1/51, 1.96%, CI (0.05–10.5) by both criteria) and PIs (1/51, 1.96%, CI (0.05–10.5) according to the CPR tool only). Simultaneous resistance to NNRTI and NRTI was observed in 1/51 (1.96%, CI (0.05–10.5)) patients.

Both CPR tool and IAS-USA algorithms were concordant in identifying five NNRTIs resistance-associated mutations: K103N in one patient (1.96%), Y188L, and H221Y in another patient (1.96%), K101E in one patient (1.96%), and V106A in another patient (1.96%). However, L234I; which is detected in one individual (1.96%), was recognized by the CPR tool only (Table 2). On the contrary, E138A mutation, which is associated with reduced RPV and ETR susceptibility, was detected by the IAS-USA mutation list only in two individuals (3.92%) (Table 2).

**Table 2:** Pretreatment drug resistance mutations detected and their resistance pattern to common drugs

Sample ID	Age/Sex	Viral load (copies/ml)	Mutations type					
			NNRTIs	Resistant to	NRTIs	Resistant to	PIs	Resistant to
MT416676	32/F	134,263	None		None		G73S*	ATV/ra
MT416698	37/F	124,246	**K103N**	EFV^c^, NVP^c^	None		None	
MT416672	45/M	19,652	Y188L, H221Y, L234I*	ETR^a^, DOR^c^, EFV^c^, NVP^c^, RPV^c^	**Y115F, M184V**	ABC^c^, FTC^c^, 3TC^c^	None	
MT416680	40/M	443,919	**K101E**	ETR^a^, EFV^a^,NVP^b^, RPV^b^, DORa	None		None	
MT416667	25/F	66,791	**V106A, E138A**	ETR^a^, DOR^c^, EFV^b^, NVPc, RPVa	None		None	
MT416710	27/F	14,498	*E138A*	ETR^a^, RPV^a^	None		None	

*F* Female, *M* Male, *ATV/r* Atazanavir/ritonavir, *ABC* Abacavir, *FTC* Emtricitabine, *3TC* Lamivudine, *DOR* Doravirine, *EFV* Efavirenz, *ETR* Etravirine, *NVP* Nevirapine, *RPV* Rilpivirine, *NRTI* nucleoside reverse transcriptase inhibitor; *NNRTI* non-NRTI, *PI* protease inhibitor

^a^Low-level resistance, ^b^Intermediate-level drug resistance, ^c^High-level drug resistance

Mutations in bold, are PDRMs by both IAS-USA and the CPR tool; Mutation in Italics are reported by IAS-USA only. While indicated by * only detected by the CPR tool

Y115F and M184V PDR mutations that are recognized by both criteria, which confer limited resistance against NRTI drugs (abacavir, emtricitabine, lamivudine), were observed in one patient (1.96%) (Table 2). G75S PDR mutation that confers low-level resistance to PI drug class atazanavir/ritonavir was observed in another study participant according to the CPR tool only (Table 2).

**Fig. 1:**
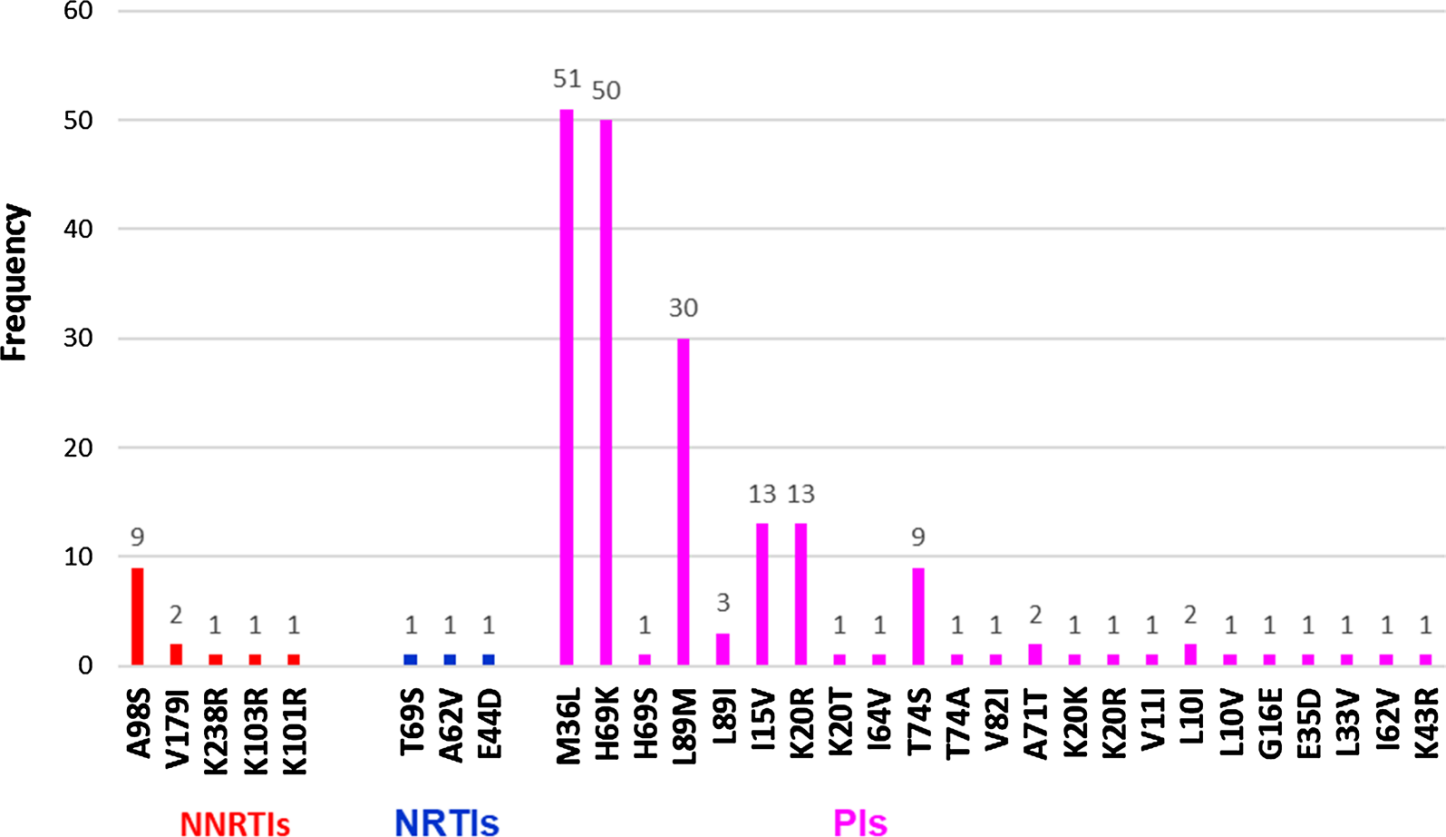
Minor drug-resistant mutations and/or polymorphisms

In addition to the above-mentioned major mutations, several additional minor drug-related mutations and/or polymorphisms recognized by both algorithms were also observed on the RT and PR of all sequenced samples (Fig. 1). Single accessory resistance mutations and/or polymorphisms were present in over 15.6% (n = 8) of RT sequences with A98S (17.6%) and V179I (3.9%) being the most frequently observed mutations, while all PR sequences harbored at least two minor PIs resistance mutations and/or polymorphisms (Fig. 1). The most frequent mutations observed in the PR sequence were H69K (100%) and M36L (98%), followed by L89M (58.8%), I15V (25.5%), K20R (25.5%), T74S (17.6%) and L89I (5.88%) (Fig. 1).

### HIV-1 genetic diversity

**Fig. 2:**
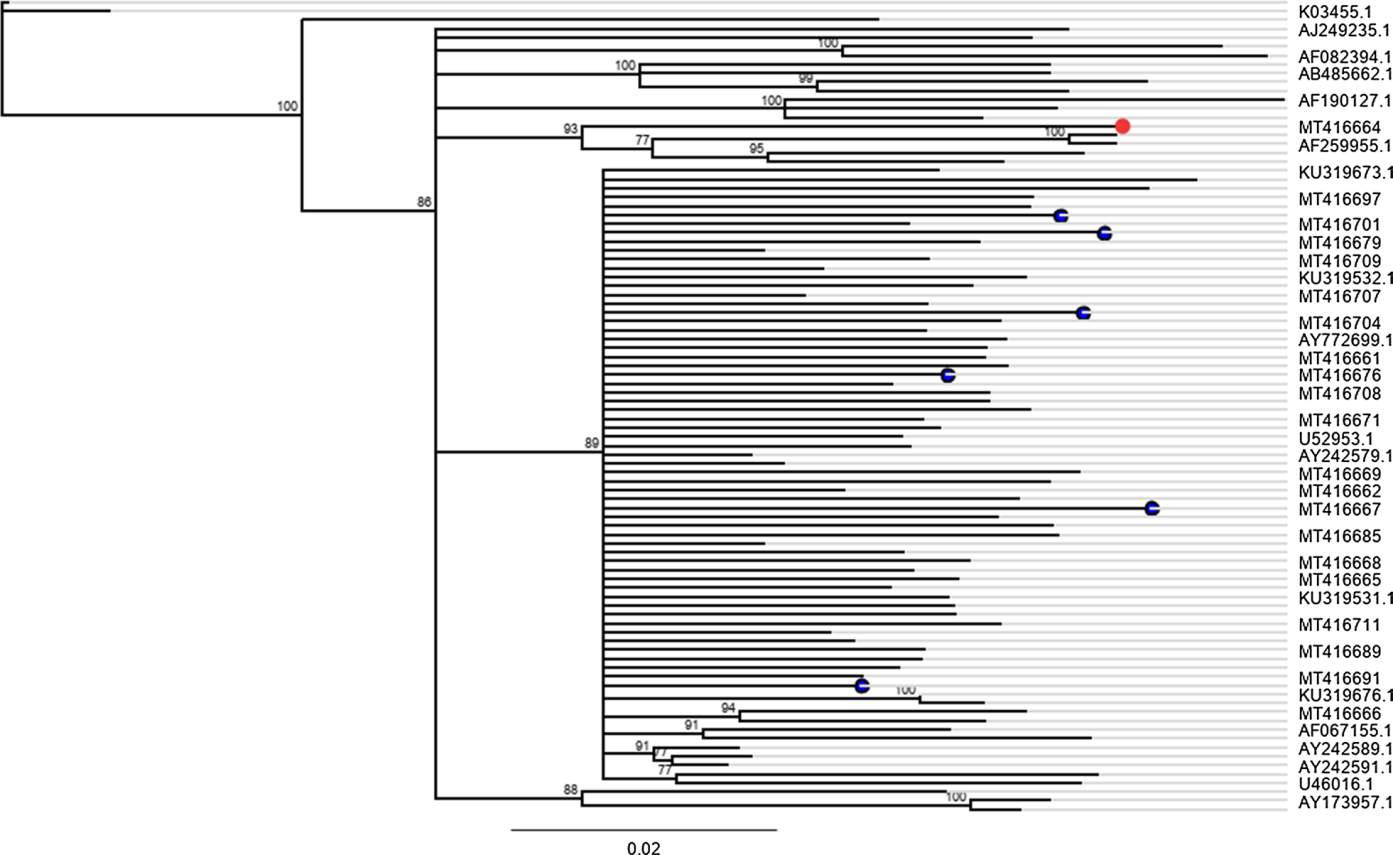
Neighbor-joining phylogenetic tree of the pol gene (PR and RT nucleotide sequences) of 51 samples along with reference sequences from the Los Alamos HIV database (http://hiv-web.lanl.gov). Those with GenBank accession number MT416661–MT416711 are sequences from this study and sequences with PDR mutations are highlighted with a blue node while one (Subtype A1) is highlighted with a red node. The rest are HIV-1 reference sequences retrieved from the Los Alamos HIV database. Reference sequences: U46016, AY242579–AY242597, KU319672–KU319798 (C-Ethiopia), AY772699 (C-S. Africa), AF067155 (C-India), U52953 (C-Brazil), AX149771 (BC-China), and the rest reference sequences are non-C. Only bootstrap values > 70% are indicated at each node

With regard to HIV-1 genetic diversity, phylogenetic analysis using the REGA HIV-1 subtyping tool (https://www.genomedetective.com/app/typingtool/hiv). showed that 98% (50/51) of the study participants were infected with HIV-1C virus while one individual (2%: 1/51) was infected with the HIV-1A1 virus. These results were further confirmed using reference sequences retrieved from the Los Alamos HIV sequence database (http://hiv-web.lanl.gov) (Fig. 2). All except one (MT416664; subtype A1, which clustered with subtype AE reference sequence with a bootstrap value of 93%) were clustered with HIV1C reference sequence with a bootstrap value of 89% (Fig. 2). Based on the phylogenetic analysis eight closely related transmission clusters with a bootstrap value of ≥ 99% were observed (Fig. 2).

## Discussion

Drug resistance poses a challenge for viral suppression, which in turn jeopardizes prevention plan against HIV infection [22]. In this study, we sequenced the partial pol gene from supposedly ART naive HIV infected individuals to show the current viral diversity and the rate of Antiretroviral Drug (ARV) resistance mutations in Addis Ababa, Ethiopia. The finding of this study indicates that 98% (50/51) of the study participants were infected with HIV-1C virus while one individual (2%) was infected with HIV-1 A1 virus. In agreement with previously published studies [16, 17], this finding indicated that the HIV-1C virus still dominates the HIV-1 epidemic in Ethiopia. Therefore, this is the latest evidence that showed persistence HIV-1C clade homogeneity in the country.

**Fig. 3:**
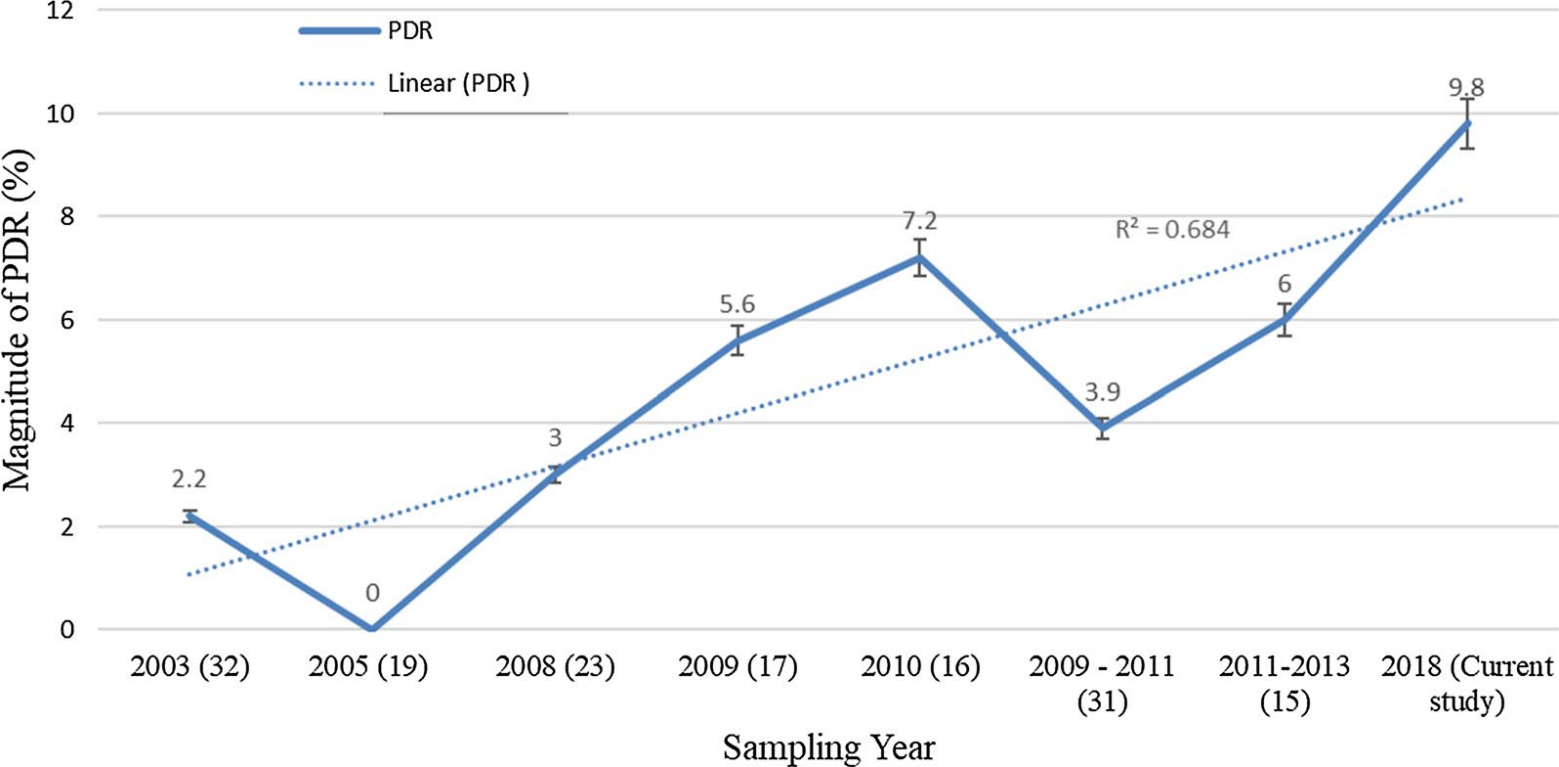
Trend of PDR Magnitude over years in Ethiopia. The numbers in parenthesis are meant to indicate reference numbers from which the data were extracted

WHO categorizes PDR into three: low (< 5%), moderate (5–15%) or high (> 15%) categories [21]. Based on this PDR classification, this study revealed that the prevalence of PDR is moderate (9.8%, CI (3.3–21.4)) in the study area but with an increased level of resistance albeit few sample size (wide CI). In contrast to this finding, low levels of PDR among pregnant women [19] and ART-naive adult individuals [23] were reported previously from the same study area. Similarly, a study done in Brazil among ART-naive pregnant women indicated a lower PDR detection rate [24]. In addition to the above previously published data from Ethiopia, this finding is in agreement with studies done in India [25], Brazil [26–28], China [29], and Iran [30], which reported a moderate magnitude of PDR among HIV-1 antiretroviral adults. The absence of PDRM screening before starting ART and poor level of adherence might have contributed to this increment of PDR in the current study. In agreement with the finding of this study, a moderate level (5.6–7.2%) of PDR among HIV-1 ART-naive individuals was reported from Northern Ethiopia [15–17]. This study, combined with all the above previously published reports in Ethiopia, showed a moderate level (but slowly increasing) level of PDR 15 years after the rollout of ART in the country (Fig. 3).

Similar to previously published studies in Ethiopia [16, 23], NNRTI-associated mutations were the most common mutations observed relative to other major mutations (NRTI and PI-associated). However, the frequency of these NNRTI-associated mutations observed in this study (7.8%) was significantly higher compared to reports from previous PDR studies done in Ethiopia (1.5–3.6%) [16, 23, 31], except for the moderate (6%) frequency reported from Gondar [15]. This increased PDR in the current study might be due to the absence of drug resistance testing before ART initiation and consecutively high transmission rate of viruses harboring resistance-conferring mutations.

The lower frequency of NRTI and PI associated mutations observed in this study are in agreement with previously conducted studies among ART-naive individuals in Ethiopia [16, 17, 23, 31, 32]. Likewise, similar NRTI and PI associated PDRMs were reported in other countries like Brazil [26] and India [25].

**Fig. 4:**
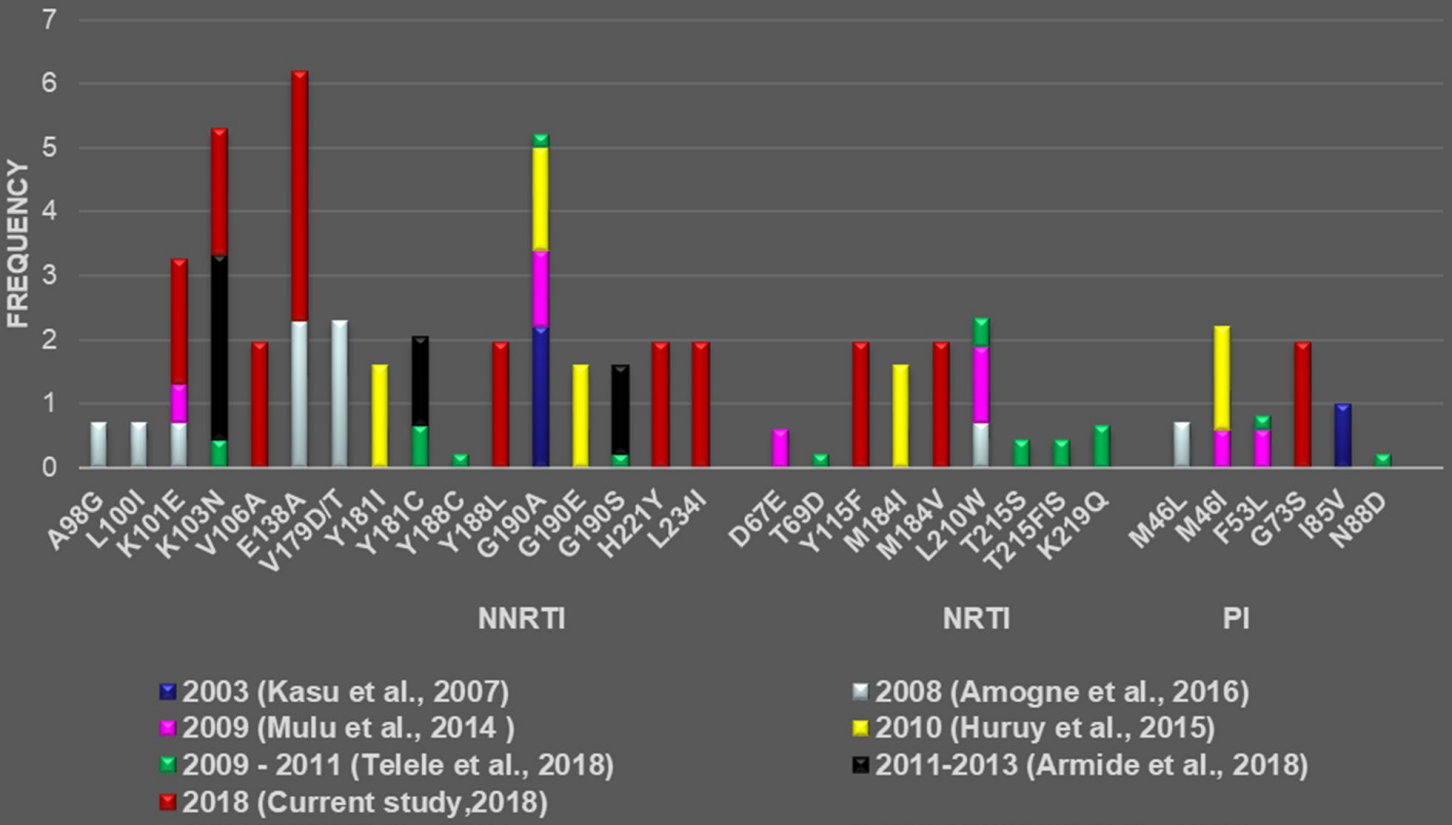
Comparison of major primary drug resistance mutations identified in treatment-naive Ethiopian individuals infected with HIV-1C

In agreement with our report, K101E, K103N, and E138A mutations of the RT region had been reported previously in Ethiopia among ART-naive individuals [17, 23, 31]. On the other hand, V106A (Mutation in the RT region that confers resistance to NNRTIs), M114V and Y115F (Mutations in the RTs region that confer resistance to NRTIs) and G73S (Mutation in the PI region that confers resistance to PIs) were all detected for the first time (Fig. 4) among ART-naive individuals in Ethiopia. The sources of these mutations could be transmission from individuals on ART already failing treatment since they are likely to be engaged in a risky sexual activity. The other possibility could be due to a natural variation in subtype C, which might have led to the emergence of these resistance conferring mutations. Although they are seen for the first time among ART-naive individuals in the country, these mutations were reported among ART-naive individuals from other countries [33].

E138A mutation, which is associated with a decreased response to ETR and RPV according to the IAS-USA mutation list, was found in 3.9% (2/51) of the specimens showing a similar frequency of detection in previous reports from Ethiopia [17]. In agreement with the previous report from Gonder [16], one individual had PDR mutations associated with two combined drug classes (NRTIs plus NNRTIs).

With regard to minor mutations and/or polymorphisms in this study, a high rate of polymorphic change was observed most frequently in the PR region than in the RT region. This, the so-called accessory mutations or resistance-related mutations, may not reflect PDR but may represent natural HIV-1 genetic variability with possible clinical implications if present with other mutations. The most frequent minor mutations observed in the PR sequence were H69K (100%) and M36L (98%), followed by L89M (58.8%), I15V (25.5%), K20R (25.5%), and T74S (17.6%). These mutations were reported with relatively the same frequencies from another study in Ethiopia [17] and India [34]. The presence of these mutations indicates a natural variation in HIV-1C virus across the globe.

## Conclusions

In conclusion, this study presents additional evidence on the HIV-1C clade homogeneity after three decades of HIV-1 circulation in Ethiopia. The study also showed an increased level of HIV-1 PDR 15 years after the rollout of ART in Ethiopia. Therefore, high follow-up and counseling strategies are warranted to those who are experiencing virological failure to prevent further dissemination of drug resistant viruses.

In general, this study showed increased levels of HIV-1 PDR, with NNRTIs associated mutations being the most frequently detected in Ethiopia that potentially compromise the effectiveness of ARV drugs especially those based on NNRTI regimens. This highlights the need for routine HIV-1 drug resistance testing before initiation of ART, and a broader public health action to prevent the emergence and transmission of drug-resistant variants. In addition, consistent follow-up and strengthening of adherence patterns, and robust monitoring of viral load to identify early treatment failure is warranted for successful ART programs and overall prevention of HIV transmission in the country and to support the global efforts in achieving the third 90 of the UNAIDS target.

## Data Availability

Sequences from this study can be found from GenBank. (Have been given Accession Number: MT416661–MT416711).

## Abbreviations

AHRI: Armauer Hansen Research Institute
AIDS: Acquired Immuno Deficiency Syndrome
ART: Antiretroviral treatment
ARV: Antiretroviral drugs
EDTA: Ethylene diamine tetra acetic acid
HIV: Human Immunodeficiency Virus
NRTI: Nucleoside reverse-transcriptase inhibitor
NNRTI: Non-nucleoside reverse-transcriptase inhibitor
PCR: Polymerase chain reaction
PDR: Pretreatment drug resistance
PDRM: Pretreatment drug resistance mutation
PI: Protease inhibitor
PR: Protease
RT: Reverse transcriptase
